# From social cognition to consumption: toward an EEG-based approach to consumer mentalizing

**DOI:** 10.3389/fpsyg.2026.1740012

**Published:** 2026-04-24

**Authors:** Chiara Casiraghi, Margherita Zito, Vincenzo Russo

**Affiliations:** 1 Behavior and Brain Lab IULM–Neuromarketing Research Center, Università IULM, Milan, Italy; 2 Department of Business, Law, Economics and Consumer Behavior “Carlo A. Ricciardi”, Università IULM, Milan, Italy

**Keywords:** consumer behavior, consumer neuroscience, EEG, mentalizing, neuromarketing, social cognition, theory of mind, mental state attribution

## 1 Introduction

Social cognition encompasses the mental processes that enable individuals to perceive and understand the mental states, emotions, and behaviors of themselves and others, allowing for appropriate social interactions (Blume et al., 2015). Among the various social cognition components, *mentalizing* refers to those higher-order cognitive abilities to infer one's own and others' mental states, being one of the most comprehensive and fundamental social cognition processes (Quesque et al., 2024). Since it underlies everyday social interactions, mentalizing plays a central role across all contexts involving social components. It is also highly relevant in consumer behavior, where many decisions are shaped by social cognition mechanisms, from product/brand identification, reputation, to imitation, social norms, and influencer dynamics. While marketing research has progressively shifted toward the consumers and the factors shaping their behavior, it still focuses on basic processes such as emotion, attention, or memory, neglecting more complex mechanisms such as mentalizing. In this opinion paper, we discuss the importance of mentalizing to better assess consumer behavior, particularly in social contexts, and propose that neuroscientific methods, especially electroencephalography (EEG), can provide effective tools for measuring this complex yet fundamental process.

## 2 Mentalizing: from social cognition to consumer behavior

Mentalizing describes the ability to infer and represent one's own and others' internal states, beliefs, intentions, and emotions (Quesque et al., 2024). It allows individuals to move beyond simple observable behavior and interpret its underlying mental causes (Van Overwalle, 2011), constituting a core social cognition process. Indeed, neuroimaging studies show that mentalizing engages a series of areas involved in higher-level social cognitive and affective processing (Lieberman et al., 2019), which are not recruited during basic reasoning.

Despite its increasing relevance, the term mentalizing has often been used interchangeably with related constructs (i.e., Theory of Mind, empathy, perspective-taking), which has contributed to conceptual ambiguity in the literature and hindered a clear understanding of its role. Recent consensus (Quesque et al., 2024), sought to better define these relationships, positioning mentalizing as the more fundamental inferential process through which individuals attribute mental states to themselves and others. Within this framework, mentalizing is differentiated from empathy–more complex and involving emotional resonance and affective sharing (Kanske et al., 2015). It is also distinguished from Theory of Mind, which refers to the use of societal heuristics to interpret others' behavior - although the two terms have frequently overlapped in prior research - and from perspective-taking, which involves cognitively adopting another person's point of view (Quesque et al., 2024).

Following this consensus perspective, we adopt the term mentalizing to refer to the core process underlying mental state attribution. This conceptualization is particularly relevant for consumer research, as consumption contexts frequently require individuals to infer the intentions, motivations, and emotions of social agents. Importantly, here we conceptualize mentalizing not as a stable individual trait, but as a social-cognitive *process* emerging during consumption contexts and interactions (Van Overwalle, 2011; Quesque et al., 2024).

Given its core role in everyday interpersonal exchanges, mentalizing has gained increasing academic attention for its relevance across a wide range of contexts. Describing these contexts is beyond the scope of this paper, which concentrates on one domain where mentalizing has not been sufficiently considered: consumer research.

In consumer contexts, mentalizing provides a powerful framework for interpreting how individuals behave. When observing others in a consumption context (e.g., watching another consumer purchasing a product, an ambassador endorsing a brand, an actor interacting with a product in an advertisement), individuals may not merely perceive the scene, but also infer the other person's emotional states, goals, and intentions. Then, such mentalizing processes may facilitate identificatory responses toward the other person and to what the other person is consuming, forming stronger attitudes toward the product (De Graaf et al., 2011) and higher message acceptance (Wojcieszak and Kim, 2016). In nowadays consumer contexts, this mechanism is not limited to direct observation, but extends to mediated consumption forms, specifically social media interactions with influencers, testimonials, or characters in advertisements and TV content (Fiolić and KneŽević, 2024), where consumers may mentalize with others even without physical copresence. Mentalizing also appears to be closely linked to processes of social conformity, where consumers align their choices with social norms, reputation, or others' evaluations - a series of well-documented social psychology phenomena including observational learning (Bandura, 1977), social comparison (Festinger, 1954), and social proof (Powell et al., 2017). Moreover, recent evidence from *consumer neuroscience–*the discipline employing neuroscientific methods to study consumer behavior (Plassmann et al., 2015)–strengthens this view, with functional magnetic resonance imaging (fMRI) research showing that advertisements engaging the mentalizing network lead to higher liking (Chan et al., 2023) and message effectiveness (Scholz et al., 2025).

Given the relevance of other discussed social processes and contexts, it is important to acknowledge that several consumer research streams implicitly involve similar mental state inference. For instance, studies on narrative persuasion mention mechanisms of identification with characters or mental simulation of situations (Green and Appel, 2024), which inherently rely on the ability to infer others' cognitions and emotions. However, these processes are rarely explicitly framed in terms of mentalizing. This lack of conceptual integration may have limited a more comprehensive understanding of the social-cognitive mechanisms underlying consumer responses.

In any case, all this evidence - from social cognition work to the related consumer research applications - highlights the importance of mentalizing and emphasizes that consumer behavior cannot be fully explained by emotional or cognitive mechanisms alone: understanding how and when consumers engage in mentalizing is fundamental to capturing the socially embedded nature of consumption. To this end, a methodological approach capable of accurately assessing the dynamics of mentalizing processes within consumption contexts is required.

## 3 How to measure mentalizing? The advantages of EEG

Assessing mentalizing is inherently challenging, as it is a multidimensional process encompassing cognitive and affective components as well as social and physical factors, operating both implicitly and explicitly (Achim et al., 2013). Traditional mentalizing assessment (e.g., Abell et al., 2000; Baron-Cohen et al., 2001), largely developed within clinical and developmental psychology, cannot be generalized to non-clinical populations and to consumption settings. Moreover, mentalizing scales (e.g., Hausberg et al., 2012; Dimitrijević et al., 2017; Gori et al., 2021; Stefana et al., 2024) not only have been mainly developed within clinical contexts, but–like all self-report measures–are prone to bias. Neuroscientific methods could address this issue, providing access to mentalizing dynamics that traditional tools cannot reach. But even these techniques present their limitations. Although fMRI has mapped the neural architecture of mentalizing (Schurz et al., 2014; Wang et al., 2021), its poor temporal resolution and immobile setup make it unsuitable for capturing the interactive features of mentalizing, constrain its applicability to consumer experiences, and limit its transferability from academia to applied research. Moreover, eye-tracking, the consumer neuroscience tool most frequently used in practice (Casado-Aranda et al., 2023), does not provide direct access to complex social-cognitive processes like mentalizing. Other techniques, such as functional near-infrared spectroscopy (fNIRS), are less expensive, portable, and tolerant to motion, but limited by shallow spatial resolution and related interpretative challenges (Casado-Aranda et al., 2023). Similarly, autonomic measures (i.e., galvanic skin response, heart rate) can be useful for tracking consumers' affective responses (Bilucaglia et al., 2025), but cannot isolate the cognitive and inferential features of mentalizing.

Against this, EEG emerges as a promising approach. Its millisecond-level temporal resolution enables a precise tracking of mentalizing dynamics as they unfold, providing a real-time view of how consumers infer others' mental states. It is more portable, cost-effective, easily adaptable to diverse scenarios (from video advertisements to purchase interactions and social media dynamics), and it is adopted not only in academia but also in applied market research (McInnes et al., 2022). All these qualities make EEG particularly well-suited for further exploration aimed at developing new measures of consumer responses (Kolar et al., 2021). When paired with robust, ecologically valid paradigms, it can support reliable assessment of mentalizing and, more generally, of cognitive-emotional processes underlying consumer behavior.

While these features provide clear advantages for EEG, it is also important to acknowledge its limitations and challenges involved in assessing complex processes like mentalizing. First, EEG is sensitive to motion artifacts and external noise, which may constrain its use in realistic settings. Moreover, its interpretation is inherently complex and challenges construct validity: EEG signals can often reflect multiple overlapping processes, making it difficult to attribute specific EEG measures exclusively to mentalizing. For instance, while some evidences suggest the role of theta band EEG activity, the same theta activity has also been associated with memorization and cognitive load processes (Fici et al., 2024).

Despite these challenges, the available EEG evidence–still limited and fragmented–provides some initial indications of the patterns that may be expected. For instance - as noted above - prior studies suggest a potential role of theta activity (Blume et al., 2015), particularly in relation to alpha activity (Imperatori et al., 2017). However, the evidence is inconsistent: for example, Yun et al. (2024) report a role of alpha activity. Such discrepancies highlight both the scarcity and heterogeneity of current findings, underscoring the need for further research and making it difficult, at present, to formulate precise hypotheses.

## 4 Integrating mentalizing into consumer neuroscience assessments

**Figure 1:**
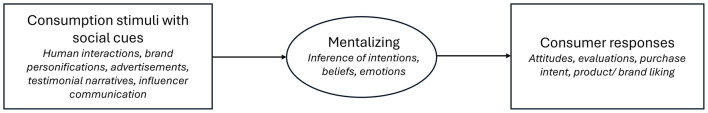
Conceptual framework illustrating the mediating role of mentalizing in consumer behavior. Consumption stimuli with social cues elicit mentalizing processes, which in turn shape consumer responses.

Building on the evidence discussed so far, we propose that mentalizing should be regarded as a core process *mediating* the relationship between marketing stimuli containing social cues (e.g., human interactions, brand personifications, advertisements, testimonial narratives, influencer communication) and consumer responses (Figure 1).

*Theoretically*, this perspective can be integrated within established consumer research frameworks, such as persuasion and dual-process models (e.g., Elaboration Likelihood Model, Heuristic–Systematic Model), which explain persuasion as depending on cognitive resources and the use of affective heuristic responses. While these frameworks have traditionally focused on emotional and cognitive responses (e.g., valence, arousal, attention, engagement, memory), incorporating mentalizing highlights an additional social-cognitive layer through which consumers interpret others' behaviors within consumption contexts. This perspective is also consistent with communication models such as Narrative Transportation Theory (Green and Brock, 2000), highly relevant in advertising and influencer communication, where characters and narratives may elicit mentalizing processes that mediate the relationship between narrative exposure and persuasive outcome.

To *practically* investigate mentalizing, its complexity requires moving beyond traditional approaches and adopting consumer neuroscience methods, with EEG-based paradigms offering promising research opportunities. EEG can be used to study consumer interactions (participants observing others engaging with products or brands), allowing researchers to capture the EEG features of consumers' mentalizing processes as they unfold. A similar approach can be applied to advertising research, where participants are exposed to narrative commercials in which characters play a central role and may elicit mentalizing responses, potentially integrating and clarifying the mechanisms of the Narrative Transportation Theory (Green and Brock, 2000), or to influencer content designed to evoke mentalizing processes. Decision-making paradigms (e.g., adapted versions of the Ultimatum Game) can be employed to assess how consumers infer emotions, intentions, and reciprocity within market-like exchanges. Hyperscanning approaches could also be implemented by simultaneously recording the EEG activity of two interacting consumers, or other pairs (e.g., influencer and follower). Finally, these mentalizing-related insights can help improve communication strategies and overall consumer engagement across marketing contexts.

Despite its potential, this approach still faces several methodological challenges that must be addressed before this research stream can advance. Beyond the already discussed limitations inherent to EEG itself, additional constraints emerge considering the specific methods. For instance, the use of hyperscanning and interactive paradigms introduces further challenges, as these setups require precise synchronization across participants, increasing technical complexity and reducing scalability, and they often involve higher costs, longer times, and specialized experts, making them less feasible for everyday marketing applications.

To address these challenges and develop a specific consumer mentalizing EEG measure, consumer neuroscience researchers should first develop EEG-based measures of mentalizing, building on established paradigms in which the degree of mentalizing required is systematically manipulated e.g., social video-based tasks such as the animated shaped of Abell et al., 2000, or more realistic tasks requiring participants to infer others' mental states (e.g., Baron-Cohen et al., 2001; Dziobek et al., 2006; Spiers and Maguire, 2006; Golan et al., 2006) - and formulating hypotheses and analyses methods based on initial EEG evidence (e.g., Cohen et al., 2008; Blume et al., 2015; Imperatori et al., 2017; Yun et al., 2024), which so far remain limited, inconsistent, and non-transferable to consumer contexts. These paradigms could first be tested in controlled laboratory settings and then progressively extended to more naturalistic environments. EEG recordings could be collected during task performance, alongside self-report measures of mentalizing extent for data triangulation and a more robust interpretation of results.

Then the results should be adapted to paradigms that balance ecological validity and experimental control, starting, for example, from the structure of already existing mentalizing tasks, adapting them to consumer-relevant stimuli and ensuring compatibility with EEG recording. High-quality EEG data is a crucial methodological requirement. Initial investigations should rely on high-density systems (around 50–60 electrodes) with standard electrode configurations (e.g., 10–20 system) to achieve sufficient spatial resolution for a more precise assessment of the neural processes involved in mentalizing. Moreover, these EEG measures should be combined with behavioral and explicit assessments to relate EEG data with well-known and observable consumer responses–particularly considering the discussed construct validity challenges. Such mentalizing EEG measures would then be validated within carefully designed paradigms that manipulate social cognition while controlling for other variables such as cognitive load, attention and emotional responses. Rather than relying on a single EEG measure, an approach integrating EEG data, behavioral responses, and self-reports may provide a more reliable and valid assessment of mentalizing in consumer contexts. Over time, such measures could be systematically validated and standardized, enabling comparability across studies and contributing to the development of robust EEG-based indicators of mentalizing in consumer research, with significant implications for both academic research and marketing practice. In terms of signal processing, both spectral and functional connectivity approaches should be considered. While the first assesses brain activity within specific frequency bands, connectivity measures (e.g., MST-based approaches) can capture the coordination between distributed brain regions, which could be particularly relevant for complex social-cognitive processes such as mentalizing.

Finally, it is important to acknowledge the *ethical implications* of measuring processes such as mentalizing in consumer research. These approaches may raise concerns related to psychological privacy, as they aim to capture how individuals infer others' intentions, beliefs, and emotions. Moreover, the application of these insights in marketing contexts could raise concerns about potential manipulation, as they could be used to design persuasive strategies in ways that are not fully transparent to consumers. This would require careful attention to informed consent, transparency, and data protection, with a strict adherence to established ethical guidelines in both neuroscience and consumer research for a responsible and non-exploitative use.

## 5 Discussion

In this paper, we propose mentalizing as a key yet overlooked consumer behavior component. Drawing from social cognition research, we discuss how the ability to infer others' mental states–both cognitions and emotions–could play a significant role in shaping how consumers interpret, engage with, and respond to a wide range of marketing messages. This becomes even more relevant in socially rich contexts such as advertising showing human interactions, influencer marketing, or interpersonal exchanges during sales. In these cases, consumers not only respond to the message content but also infer others' emotions and intentions, which in turn could influence their evaluations, attitudes, and decisions.

Despite its theoretical importance, mentalizing has rarely been considered within marketing and consumer research. A major reason for this gap lies in the methodological difficulty of capturing such a multidimensional and dynamic process in an ecologically valid way. Traditional methods, such as questionnaires and laboratory-based tasks developed in clinical psychology, are only limitedly informative and not suited for consumer contexts. Neuroscientific techniques, particularly EEG, may represent a promising solution to these limitations. Thanks to its high temporal resolution and flexibility, EEG could enable the study of mentalizing dynamics as they occur in consumption scenarios, such as video advertisements or social media interactions. In any case, both other neuroscientific and traditional methods should not be seen in opposition: a multimethod approach, combining EEG with other measures, could offer a more complete picture, capturing both the *what* (e.g., product liking) and the *why* (e.g., mentalizing) behind consumer responses.

Future research should aim to adapt established mentalizing paradigms for EEG implementation, validate them in non-clinical populations and within consumer samples, and test their value for relevant marketing outcomes. Developing standardized EEG measures for mentalizing will be an important step toward methodological robustness, comparability across studies, and final applicability.

Integrating mentalizing into the “set” of consumer neuroscience measures along with behavioral and self-report measures, can enrich our understanding of the social-cognitive foundations of consumer behavior, particularly how individuals perceive and interpret others within consumption contexts. In this perspective, EEG represents a promising complementary tool rather than a standalone solution for a more effective, person-centered consumer research. Finally, this research agenda requires careful consideration of the ethical implications associated with measuring mentalizing processes, ensuring that such approach is developed and applied in a transparent, non-exploitative, and responsible manner.
